# Longitudinal associations of psychological distress with subsequent cognitive decline and dementia: a multi‐cohort study

**DOI:** 10.1002/alz.71093

**Published:** 2026-03-08

**Authors:** Jean Stafford, Serhiy Dekhtyar, Tom C Russ, Archana Singh‐Manoux, Jane Maddock, Kate Walters, Vasiliki Orgeta, Neil Davies, James B Kirkbride, Marcus Richards, Robert Howard, Praveetha Patalay

**Affiliations:** ^1^ Advanced Care Research Centre Usher Institute, College of Medicine and Veterinary Medicine, University of Edinburgh Edinburgh UK; ^2^ Unit for Lifelong Health and Ageing at UCL Faculty of Population Health Sciences University College London (UCL) London UK; ^3^ Aging Research Center Department of Neurobiology, Care Sciences and Society Karolinska Institutet, and Stockholm University Stockholm Sweden; ^4^ Alzheimer Scotland Dementia Research Centre University of Edinburgh Edinburgh UK; ^5^ Division of Psychiatry Centre for Clinical Brain Sciences University of Edinburgh Edinburgh UK; ^6^ Division of Psychiatry Faculty of Brain Sciences, UCL London UK; ^7^ Inserm U1153 Epidemiology of Ageing and Neurodegenerative Diseases Université Paris Cité Paris France; ^8^ Division of Surgery and Interventional Science London UK; ^9^ Research Department of Primary Care and Population Health, Faculty of Population Health Sciences UCL London UK; ^10^ Centre for Longitudinal Studies UCL Institute of Education, UCL London UK

**Keywords:** cognition, dementia, epidemiology, mental health, multi‐cohort, psychological distress

## Abstract

**INTRODUCTION:**

Psychological distress has been linked with cognitive impairment. However, whether the relationship is causal, reflects preclinical dementia neuropathology, or confounding by common causes remains unclear.

**METHODS:**

In five UK longitudinal studies, we examined associations of psychological distress with subsequent cognition using linear and mixed effects models, and dementia using logistic regression. We examined variation by age‐at‐assessment, severity, and distress persistence, combining study‐specific estimates using two‐stage individual participant data meta‐analysis.

**RESULTS:**

Pooling across studies (N = 24,564), greater baseline psychological distress was associated with lower subsequent cognitive level (β = −0.03 [95% confidence interval [CI]: −0.06; −0.01]; *I* ^2^ = 70%), and dementia (odd ratio [OR] = 1.1 [1.0; 1.2]; *I* ^2^ = 0%), but not cognitive change. Associations were found for clinically significant, persistent and intermittent distress. Dementia was associated with distress assessed at ages 65‐75, and 55‐64, but not 45‐54 years.

**DISCUSSION:**

Findings highlight the relevance of psychological distress in later cognitive outcomes, with potential future implications for dementia prevention and identifying high‐risk groups.

## Highlights

- Psychological distress was associated with lower cognitive level in five UK studies.
- Higher baseline psychological distress was associated with subsequent dementia.
- Continuous and clinically significant distress were linked with adverse outcomes.
- Persistent and intermittent distress were associated with poorer cognitive outcomes.
- Associations were found for distress at ages 55‐64 and 65‐75, but not 45‐54 years.

## 1 BACKGROUND

Experiencing mental health difficulties, such as depression, in mid‐ and late‐life has been associated with subsequent cognitive impairment and dementia.^1^, ^2^ However, few studies have examined cognitive outcomes in broader psychological distress, an umbrella term describing emotional difficulties, which can include, but is not limited to, symptoms of depression, anxiety, stress, or somatic complaints. Such symptoms may or may not be severe or longstanding enough to meet diagnostic criteria for a psychiatric disorder.^3^

In addition, temporality and the mechanisms underlying these associations are poorly understood. Gaining further insight into longitudinal relationships between psychological distress, cognition, and dementia could help to inform dementia prevention approaches, and earlier identification of high‐risk groups, both of which are major priority areas for global public health.

Most research to date has focused on cognitive outcomes in relation to specific psychiatric diagnoses or symptoms, particularly depression,^1^ which was identified as a modifiable risk factor in the Lancet Dementia commission.^4^ In contrast, studies examining broader psychological distress,^5^, ^6^, ^7^, ^8^ which often includes, but is not limited to depressive symptoms, remains relatively sparse, and has been hampered by varying assessments of distress, including single‐item measures.^9^, ^10^

Aspects of psychological distress have also shown associations with poorer subsequent cognitive functioning^11^, ^12^ and decline,^2^ although findings regarding cognitive trajectories have been mixed.^13^ For instance, in the 1946 birth cohort, affective symptoms in adolescence, but not adulthood, were associated with poorer subsequent cognitive functioning at age 43, but not with decline between ages 43‐69 years.^14^

In addition, the temporal nature of these associations is poorly understood, hence it remains unclear whether psychological distress symptoms are causal risk factors for dementia, or early markers of dementia neuropathology during the preclinical period (reverse causality). In depression, while some studies have shown associations with dementia over several decades, not all studies have shown longstanding relationships. For instance, United Kingdom and Norwegian cohort studies only found associations with depression closer to dementia diagnosis (∼11 years before),^15^, ^16^ although an association was found with anxiety 22 years before dementia in the Norwegian cohort. In addition, relatively few studies have examined temporality over long follow‐up periods in relation to wider aspects of psychological distress.^7^

Further research is also needed to examine the role of severity and persistence of psychological distress symptoms. In depression, some studies have identified associations for mild and subsyndromal symptoms,^17^, ^18^ while others only found associations with more severe symptoms.^19^, ^20^ There is some evidence that associations may vary by sex.^16^ For instance, a Norwegian population‐based study found longstanding associations between combined depression and anxiety symptoms and dementia in women 33 years before dementia diagnosis, whereas for men, no difference was found until 11 years before diagnosis.^16^ However, evidence on sex differences in relation to psychological distress and cognitive outcomes is limited, and few studies have examined variation in relation to other sociodemographic factors, such as education level.

To address these gaps, using multi‐cohort longitudinal data, we investigated longitudinal relationships between psychological distress and subsequent cognitive trajectories and dementia risk. We sought to gain further insight into the temporal nature of these relationships by using studies with long follow‐up periods and measures of psychological distress from both early‐ to mid‐ adulthood and mid‐ to late‐adulthood. We examined whether findings varied by: age of psychological distress assessment, severity and persistence of psychological distress, and sociodemographic factors including sex, education level, and occupational social class.

**RESEARCH IN CONTEXT**

1. **Systematic review**: We searched PubMed to June 19, 2025, for longitudinal, population‐based studies on psychological distress and cognitive outcomes, finding five non‐overlapping studies focused on dementia. Limitations included short follow‐up, varied distress measurement, and limited focus on symptom severity, persistence, or age‐at‐assessment.
2. **Interpretation**: Multi‐cohort analyses demonstrated that not only severe, persistent psychological distress, but also intermittent and subthreshold symptoms were associated with poorer cognitive outcomes. Findings could have important implications at the population level, given the high prevalence of mild, intermittent distress symptoms. Dementia was associated with distress assessed at ages 65‐75 and 55‐64 years, but not at ages 45‐54 years, suggesting possible reverse causality, although longstanding associations indicate that this is unlikely to fully explain findings.
3. **Future directions**: Findings could contribute to future dementia prevention models, clinical management, and identifying people at high‐risk at an earlier stage. Further research is needed to examine cognitive outcomes in relation to a wider range of psychiatric symptoms, and potential underlying mechanisms.

## 2 METHODS

### 2.1 Pre‐registration

We pre‐registered our protocol on the Open Science Framework (OSF) platform (https://doi.org/10.17605/OSF.IO/ZHG8B). Amendments to the protocol are reported in Table S1.

### 2.2 Study design and participants

We examined associations between psychological distress, cognition, and dementia using a multi‐cohort approach, and included data from five longitudinal studies (Table S2): the Caerphilly Prospective Study (CAPS), the English Longitudinal Study of Ageing (ELSA), Whitehall II (WHII), the National Child Development Study (NCDS); and the National Survey of Health and Development (NSHD). All data sources were accessed through the Dementias Platform UK (DPUK). Subsequent fluid cognition outcomes were assessed at a single time point in all datasets, and in ELSA, WHII and NSHD, we examined associations with cognitive trajectories. We examined dementia outcomes in all studies, except NCDS. For the purposes of this analysis, “baseline” refers to the first wave in adulthood at which psychological distress was assessed. This wave served as the analytic baseline for longitudinal analyses, except those examining variation by age at psychological distress assessment. Within each study, our analytical sample included participants with data on psychological distress and without prevalent dementia at baseline (where information on dementia was available at baseline); with assessments of cognition in the single time point used in analyses; and at least two cognitive assessments after baseline psychological distress assessment where cognitive trajectories were examined.

A lived experience advisory group of three former carers of people with dementia were involved throughout the study. The group provided input on study conceptualization, protocol development and interpretation of findings, and will also be involved in shaping plans for dissemination (described in Supplementary Information).

### 2.3 Psychological distress exposures

Self‐reported psychological distress at baseline was measured using validated assessment tools within each study, as reported in Table 1 and Supplementary Information. In CAPS, psychological distress was first assessed using the General Health Questionnaire‐30 (GHQ‐30) assessment in wave 1, in which participants were aged 45‐59 years. In ELSA, psychological distress was first assessed at wave 1 using the eight‐item Centre for Epidemiological Studies Depression scale (CES‐D),^21^ which measures symptoms including depressed mood, restless sleep, and decreased energy and enjoyment in life. In WHII, psychological distress was first measured in 1985, when participants were aged 35‐55 years, using the GHQ‐30,^22^ which is widely used to screen for psychiatric disorders in community samples.^21^ In NCDS, the first adulthood assessment of psychological distress was at age 23 using the Malaise Inventory,^23^ a measure of emotional disturbance and associated somatic symptoms.

**TABLE 1:** Summary of datasets and assessments.

Dataset	Baseline n at study enrollment	Baseline age at study enrollment	Psychological distress (measure, age)	Cognition (age/phase/wave)	Dementia assessed?	Psychiatric symptom severity cutoff
NSHD	5,362	Birth cohort	PSE (**age 36**); PSF (age 43); GHQ‐28 (age 53, 60—64, 69)	Memory, processing speed (age 43, 53, 60—64, 69, 77)	Age 77	PSE cutoff‐≥4 PSF cutoff‐≥23 GHQ cutoff‐≥5 (binary scoring method)
NCDS	17,415	Birth cohort	Malaise Inventory (**age 23,** 33, 42, 50, 62)	Memory, verbal fluency, processing speed and accuracy (age 50, 62)	No	Cutoff‐≥4 (9‐item version)
WHII	10,308	35‐55	GHQ‐30 (**1985‐1988**, 1989‐1990, 1991‐1994, 1997‐1999, 2001, 2002‐4, 2006, 2007‐9, 2012‐13, 2015‐16)	Verbal fluency, memory, Alice Heim 4‐I (1997‐99, 2002‐04, 2007‐09, 2012‐13, 2015‐16)	All waves	Cutoff‐≥5 (binary scoring method)
ELSA	11,931	50‐99+	CES‐D (**2002‐2003**; 2004‐2005; 2006‐2007; 2008‐2009; 2010‐2011; 2012‐2013; 2014‐2015; 2016‐2017; 2018‐2019; 2021‐2023)	Memory, verbal fluency, orientation (all waves; except verbal fluency unavailable in wave 6)	All waves	Cutoff‐≥4
CAPS	2512	45‐59	GHQ‐30 (**1979**‐**1983**; 1984‐1988; 1989‐1993; 1993‐1997)	CAMCOG (1989‐1993; 1993‐1997; 2002‐2004)	Phase 5	Cutoff‐≥5 (binary scoring method)

*Note*: Bold denotes age or wave of psychological distress assessment used as baseline assessment in primary analyses.

Abbreviations: CAMCOG, Cambridge Cognitive Examination; CAPS, Caerphilly Prospective Study; CES‐D, Center for Epidemiologic Studies Depression Scale; ELSA, English Longitudinal Study of Ageing; GHQ, General Health Questionnaire; NCDS, National Child Development Study; NSHD, National Survey of Health and Development; PSE, Present State Examination; PSF, Psychiatric Symptom Frequency scale; WHII, Whitehall II.

In NSHD, psychological distress was assessed in adulthood at the following ages: age 36 (Present State Examination),^24^ age 43 (Psychiatric Symptom Frequency),^25^ and at ages 53, 60—64, and 68—70 years (GHQ‐28).^26^ To allow comparison across waves, in line with guidance from the Centre for Longitudinal Studies (CLS) and CLOSER,^27^ we created harmonized variables based on seven conceptually similar items capturing psychological distress consistently within each measure, with items pertaining to low mood, fatigue, tense/stressed, sleep problems, panic, hopelessness, and health anxiety (Supplementary Information).

In primary analyses, the first psychological distress assessment in adulthood was used as the main exposure. We also examined associations with clinically significant psychological distress using binary variables based on validated cutoff scores on questionnaires within each study (Table 1; Supplementary Information). In addition, we examined whether associations varied depending on whether clinically significant psychological distress was observed persistently prior to cognitive assessments during the first three waves in which psychological distress assessments were available in adulthood (high symptoms in two or three waves) versus intermittently high (high symptoms in one wave only), relative to those without clinically significant psychological distress.

### 2.4 Outcomes

We computed an overall level of fluid cognition based on individual cognitive assessments of domains including memory, verbal fluency, and speed of processing. We standardized each cognitive test across timepoints and within studies on a common standard deviation‐based scale, with a mean of 0 and a standard deviation of 1. This approach facilitates interpretability over time and across studies, although we note that standardized scores reflect study‐specific distributions and are not equivalent measures across different measures and reference populations. General fluid cognitive level was computed by averaging standardized scores across individual domains and re‐standardizing the fluid cognitive level. Full information about assessments of individual cognitive domains within each study is provided in Supplementary Information.

We also examined dementia in CAPS, ELSA, WHII, and NSHD. In CAPS, at wave V (aged 68‐82 years) the Cambridge Cognitive Examination (CAMCOG),^28^ a standardized instrument to measure dementia and assess cognitive impairment, was used to select people for detailed clinical assessment, from which participants were classified as having normal cognition, cognitive impairment without dementia, or dementia. Because of the smaller sample in wave V, we included those with cognitive impairment or dementia in our outcome. In ELSA, dementia was ascertained from self‐reported physician diagnosis, with additional cases obtained from the Informant Questionnaire on Cognitive Decline in the Elderly (IQCODE), which has been validated as a sensitive screening tool for dementia,^29^ based on a validated cutoff score of 3.5 or above.^30^ In WHII, dementia diagnoses were obtained from National Health Service (NHS) Digital's Hospital Episode Statistics based on International Classification of Diseases 10th Revision (ICD‐10) codes F00, F01, F03, G30 and G31, with 78% sensitivity and 92% specificity,^31^ and from self‐reported dementia diagnosis. In NSHD, at age 77 years the AD8 interview was administered to participants’ informants to distinguish those with very mild dementia from those without dementia.^32^ The AD8 has demonstrated strong correlation with Clinical Dementia Rating Domains and performance on neuropsychological tests.^33^ Further detail about dementia ascertainment is provided in Supplementary Information.

### 2.5 Covariates

Where available across studies, models were adjusted for the following covariates at or before baseline (the first psychological distress assessment in adulthood): age (continuous in years), sex (male, female), education level (General Certificate of Secondary Education [GCSE]/O‐level or equivalent, less than GCSE/O‐level), occupational position (manual, nonmanual, other/none), marital and cohabitation status (married and/or living with a partner, unmarried and living alone), long‐term health conditions (none, one or more), smoking (current smoker, ex‐smoker, never smoked), alcohol consumption (low, moderate, high), physical activity (low, moderate, high), baseline or childhood cognition where possible (for cognitive outcomes). Full information about covariates within each study is provided in Supplementary Information.

### 2.6 Statistical analyses

We used linear regression models to examine associations between baseline psychological distress and subsequent standardized fluid cognitive level assessed at a single time point (mean ages 62‐72 years). In addition, in ELSA, WHII, and NSHD, where assessments of cognition were available in three or more time points, we used linear mixed models with random slope and intercept to examine associations between baseline psychological distress and subsequent trajectories of standardized fluid cognitive level over time, with time centered on baseline date and coded in years.

We investigated associations between baseline psychological distress and subsequent dementia in CAPS, ELSA, WHII, and NSHD using logistic regression models as the primary analyses to increase comparability across studies, as several studies assessed dementia at a single time point without information on timing of dementia. We examined whether associations with dementia varied by age of psychological distress assessment, defining three age bands for mental health exposure: ages 45‐54, 55‐64, and 65‐75 years. Within each study, we selected the psychological distress score from the assessment closest to the target age of 50, 60, or 70 years, respectively.

For all analyses, we first presented findings from models partially adjusted for age and sex only, and then maximally adjusted for available covariates from the list above. We report partially adjusted results for comparison, as while additional social and health‐related variables were conceptualized as potential confounders, it remains possible that some of these variables may also be influenced by prior psychological distress, and could therefore operate as mediators rather than confounders. We used interaction terms between psychological distress and covariates to examine whether associations varied by sociodemographic factors, including: sex in all datasets, except CAPS, where data are only available for men, education level (GCSE/O‐level or equivalent vs. less than GCSE/O‐level), and occupational social class (manual, non‐manual, other/none).

We also conducted sensitivity analyses. First, we examined associations with cognitive outcomes in relation to depressive and anxiety symptoms individually. Second, we repeated our primary analyses restricting to the three cohorts that used the GHQ to assess psychological distress (NSHD, CAPS, and WHII). Third, we re‐ran our linear mixed models including interactions between covariates and time to examine whether findings differed from our main models. Fourth, we tested for an interaction between sex, psychological distress and time to assess whether associations between psychological distress and cognitive change varied by sex. Fifth, we examined associations between baseline psychological distress and subsequent dementia using Cox regression in ELSA and WHII, where date of dementia diagnosis was available.

We analyzed data using an individual participant data two‐stage meta‐analysis approach, with study‐specific analyses completed in the first step, followed by pooling study‐specific estimates across studies where appropriate. Before analysis, we harmonised all variables between studies as far as possible (Supplementary Information). We assessed heterogeneity between estimates using the I^2^ statistic. We applied multiple imputation models using chained equations to impute missing covariate data (Table S3). All analyses were completed in Stata version 18.0.

## 3 RESULTS

Baseline characteristics of participants within each study in our analyses are presented in Table 2. A participant flow diagram is provided in Figure S1, indicating participants excluded due to having dementia at baseline, missing information on baseline psychological distress, or without information on either cognition in the single time point used in analyses or at least two cognitive assessments after baseline psychological distress assessment. Participants included in the analysis differed on socioeconomic and health‐related factors from those excluded. For instance, those excluded were more likely to have lower education, manual occupations, comorbidities, and to have negative health behaviors (Table S4). The overall analytical sample across studies was 24,564 (44.4% women; pooled mean age at baseline: 43.8 years).

**TABLE 2:** Descriptive statistics for psychological distress exposures, cognitive outcomes and covariates.

Covariates	ELSA	CAPS	WHII	NSHD	NCDS
**Overall N**	9022	1180	6599	2181	5582
**Baseline age for present study, mean (SD)**	64.1 (9.7)	51.5 (4.4)	44.3 (5.9)	36	23
**Sex**					
Male	4034 (44.7%)	1180 (100%)	4712 (71.4%)	1041 (47.7%)	2691 (48.2%)
Female	4988 (55.3%)	‐	1887 (28.6%)	1140 (52.3%)	2891 (51.8%)
**Marital status**					
Not married (or cohabiting)	1924 (21.3%)	89 (7.5%)	1553 (23.5%)	217 (9.9%)	3075 (55.1%)
Married (or cohabiting)	7098 (78.7%)	1091 (92.5%)	5046 (76.5%)	1964 (90.1%)	2507 (44.9%)
**Education**					
Less than o‐level or equivalent	3981 (44.1%)	741 (62.8%)	1805 (27.4%)	873 (40%)	1205 (21.6%)
O‐level or equivalent	4269 (47.3%)	439 (37.2%)	4794 (72.6%)	1308 (60%)	4377 (78.4%)
International/other	772 (8.6%)	‐	‐	‐	‐
**Occupational social class**					
Manual	3732 (41.4%)	715 (60.6%)	Clerical/support: 999 (15.1%)	944 (43.3%)	2135 (38.2%)
Non‐manual	5290 (58.6%)	465 (39.4%)	Professional/executive: 3349 (50.8%)	1181 (54.1%)	3447 (61.8%)
Other	‐	‐	Adminisrative: 2251 (34.1%)	56 (2.6%)	
**Long‐term health conditions**					
None	6725 (74.5%)	1021 (86.5%)	5904 (89.5%)	2103 (96.4%)	5400 (96.7%)
One or more	2297 (25.5%)	159 (13.5%)	695 (10.5%)	78 (3.6%)	182 (3.3%)
**Smoking status**					
Non‐smoker	3279 (36.3%)	239 (20.3%)	3453 (52.3%)	699 (32.0%)	1821 (32.6%)
Smoked previously	4162 (46.1%)	364 (30.8%)	2,222 (33.7%)	861 (39.5%)	1761 (31.5%)
Current smoker/smoked regularly	1581 (17.5%)	577 (48.9%)	924 (14%)	621 (28.5%)	2000 (35.8%)
**Alcohol consumption**					
Low	2635 (29.2%)	228 (19.3%)	973 (14.7%)	‐	963 (17.3%)
Moderate	3769 (41.8%)	725 (61.4%)	4456 (67.5%)	‐	3447 (61.8%)
High	2618 (29.0%)	227 (19.2%)	1170 (17.7%)	‐	1172 (21.0%)
Average units alcohol per day—mean (SD)	‐			1.8 (2.5)	‐
**Physical activity**					
Low	1999 (22.2%)	650 (55.1%)	2213 (33.5%)	756 (34.7%)	3095 (55.4%)
Moderate	4360 (48.3%)	232 (19.7%)	2741 (41.5%)	614 (28.2%)	1658 (29.7%)
High	2663 (29.5%)	298 (25.3%)	1645 (24.9%)	811 (37.2%)	829 (14.9%)
**Baseline psychological distress (binary)**					
No	7643 (84.7%)	920 (78%)	4,773 (72.3%)	1921 (88.1%)	5157 (92.4%)
Yes	1379 (15.3%)	260 (22%)	1826 (27.7%)	260 (11.9%)	425 (7.6%)
**Cognitive change—β (95% CI)**	*β* = −0.040 (95%CI: −0.042‐−0.038)	‐	*β* = −0.010 (95% CI: −0.011‐−0.009)	*β* = −0.005 (95%CI: −0.007—−0.004)	‐

*Note*: Baseline for the present study (wave of first adulthood psychological distress assessment).

Abbreviations: CAPS, Caerphilly Prospective Study; CI, confidence interval; ELSA, English Longitudinal Study of Ageing; NCDS, National Child Development Study; NSHD, National Survey of Health and Development; SD, standard deviation; WHII, Whitehall II.

### 3.1 Psychological distress and general fluid cognition

**FIGURE 1 alz71093-fig-0001:**
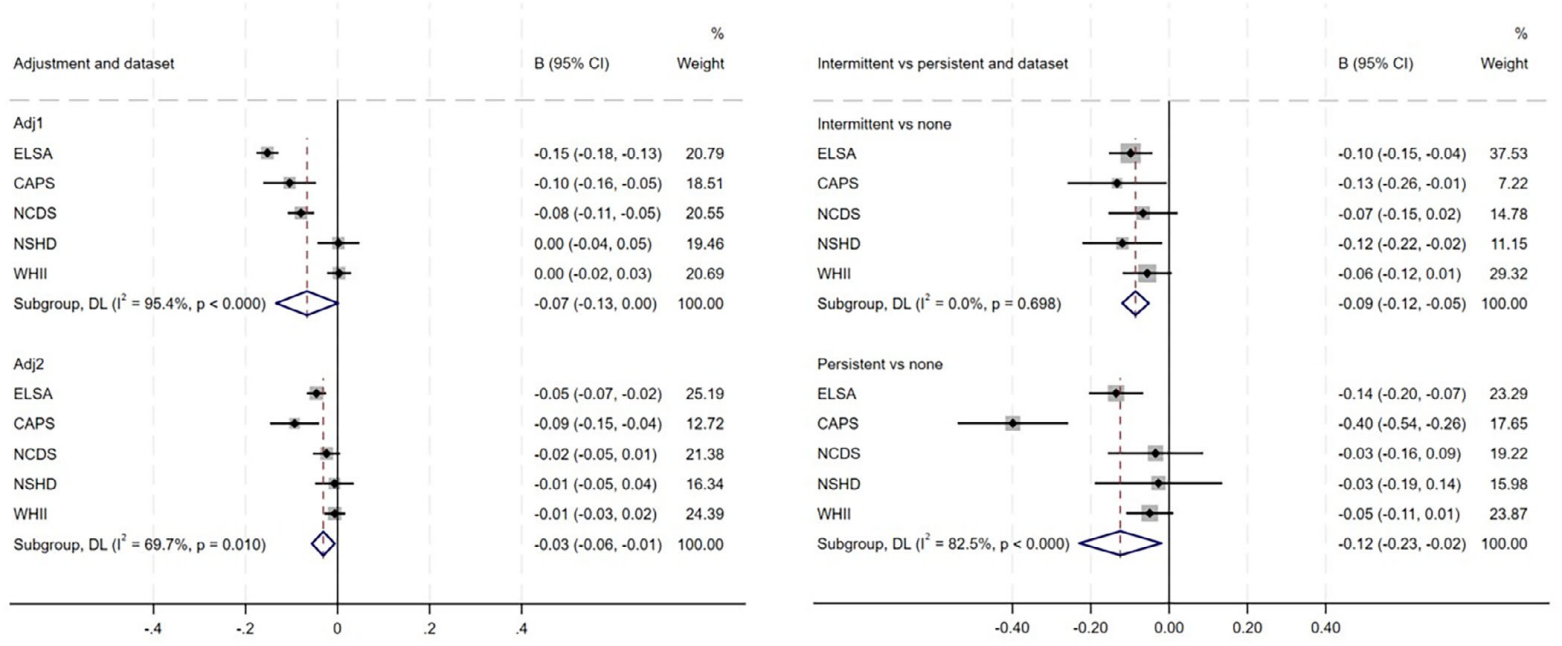
Pooled association between psychological distress and subsequent fluid cognitive levels—overall association with baseline distress (left); intermittently and persistently high versus no high psychological distress (right). β, linear regression coefficient; CI, confidence interval; CAPS, Caerphilly Prospective Study; ELSA, English Longitudinal Study of Ageing; NCDS, National Child Development Study; NSHD, National Survey of Health and Development; WHII, Whitehall II.

First, we investigated associations between baseline psychological distress and general fluid cognitive level at a single time point based on findings from linear regression models (mean ages 62‐72 years; Table 3; Figure 1; Figures S2‐S3). Pooled analyses across five studies showed that greater psychological distress was associated with poorer subsequent standardized fluid cognitive level in fully adjusted models (*β* = −0.03 [95% confidence interval {CI}: −0.06; −0.01]; *I* ^2^ = 70%). This pattern was also observed using cutoff scores indicating clinically significant psychological distress (fully adjusted model (*β* = −0.1 [−0.1; 0.0]; *I* ^2^ = 62%). Associations were found for persistent (β = −0.1 [−0.2; −0.02]; *I* ^2^ = 83%) and intermittent distress (*β* = −0.1 [−0.1; −0.1]; *I* ^2^ = 0%).

**TABLE 3:** Associations between psychological distress and cognitive outcomes.

	ELSA		WHII		CAPS		NSHD		NCDS	
Variable	Adj1 β (95% CI)	Adj2 β (95% CI)	Adj1 β (95% CI)	Adj2 β (95% CI)	Adj1 β (95% CI)	Adj2 β (95% CI)	Adj1 β (95% CI)	Adj2 β (95% CI)	Adj1 β (95% CI)	Adj2 β (95% CI)
**Standardized fluid cognitive levels (single time point)**										
Psychological distress (continuous)	−0.15 (−0.18‐0.13)*	−0.05 (−0.07‐0.02)*	0.00 (−0.02‐0.03)	−0.01 (−0.03‐0.02)	−0.10 (‐0.16‐0.05)*	−0.09 (−0.15‐0.04)*	0.00 (−0.04‐0.05)	−0.01 (‐0.05‐0.04)	−0.08 (−0.011‐0.05)*	−0.02 (−0.05‐0.01)
High symptoms vs none	−0.32 (−0.39‐0.26)*	−0.08 (−0.14‐0.03)*	0.01 (−0.05‐0.06)	−0.01 (−0.06‐0.04)	−0.25 (−0.38‐0.11)*	−0.20 (−0.33‐0.08)*	0.01 (−0.12‐0.15)	0.02 (−0.11‐0.14)	−0.17 (−0.025‐0.08)*	−0.05 (−0.13‐0.04)
Intermittent symptoms vs none	−0.22 (−0.28‐0.15)*	−0.10 (−0.15‐0.04)*	0.00 (−0.07‐0.07)	−0.06 (−0.12‐0.01)	−0.17 (−0.31‐0.04)*	−0.13 (−0.26‐0.01)*	−0.13 (−0.25‐0.02)*	−0.12 (−0.22‐0.02)*	−0.22 (−0.30‐0.14)*	−0.07 (−0.15—0.02)*
Persistent symptoms vs none	−0.41 (−0.49‐0.33)*	−0.14 (−0.20‐0.07)*	−0.03 (−0.10‐0.04)	−0.05 (−0.11‐0.01)	−0.49 (−0.64‐0.34)*	−0.40 (−0.54‐0.26)*	−0.05 (−0.22‐0.13)	−0.03 (−0.19‐0.14)	−0.27 (−0.39‐0.16)*	−0.03 (−0.16‐0.09)
**Standardized fluid cognitive trajectories (standardized rate of change per year)**										
Psychological distress (continuous)	−0.001 (−0.003‐0.001)	−0.001 (−0.003‐0.001)	0.000 (−0.001‐0.001)	−0.001 (−0.002‐0.000)	‐	‐	−0.002 (−0.005‐0.002)	0.001 (−0.004‐0.005)	‐	‐
High symptoms vs none	−0.003 (−0.009‐0.002)	−0.003 (−0.009‐0.002)	0.000 (−0.003‐0.002)	−0.001 (‐0.003‐0.002)	‐	‐	−0.003 (−0.015‐0.009)	0.004 (−0.010‐0.018)	‐	‐
Intermittent symptoms vs none	−0.002 (−0.007‐0.003)	−0.002 (−0.007‐0.003)	−0.000 (−0.003‐0.003)	0.001 (−0.002‐0.004)	‐	‐	−0.007 (−0.016‐0.002)	−0.007 (−0.019‐0.004)	‐	‐
Persistent symptoms vs none	−0.009 (−0.016‐0.003)*	−0.009 (−0.016‐0.002)*	0.002 (−0.001‐0.005)	0.002 (−0.001‐0.006)	‐	‐	0.006 (−0.010‐0.021)	0.011 (−0.008‐0.030)	‐	‐

	**Adj1 OR (95% CI)**	**Adj2 OR (95% CI)**	**Adj1 OR (95% CI)**	**Adj2 OR (95% CI)**	**Adj1 OR (95% CI)**	**Adj2 OR (95% CI)**	**Adj1 OR (95% CI)**	**Adj2 OR (95% CI)**	**Adj1 OR (95% CI)**	**Adj2 OR (95% CI)**
**Dementia**										
Psychological distress (continuous)	1.12 (1.02‐1.23)*	1.11 (1.00‐1.22)*	1.06 (0.90‐1.25)	1.05 (0.89‐1.24)	1.27 (1.07‐1.50)*	1.22 (1.02‐1.46)*	1.12 (0.95‐1.32)	1.13 (0.95‐1.34)	‐	‐
High symptoms vs none	1.40 (1.11‐1.76)*	1.36 (1.07‐1.72)*	1.04 (0.71‐1.52)	1.02 (0.70‐1.50)	1.62 (1.06‐2.48)*	1.45 (0.93‐2.25)	1.24 (0.75‐1.06)	1.20 (0.71‐2.01)	‐	‐
Intermittent symptoms vs none	1.03 (0.76‐1.40)	0.99 (0.73‐1.35)	1.46 (0.95‐2.26)	1.49 (0.96‐2.31)	1.84 (1.17‐2.91)*	1.77 (1.11‐2.82)*	1.39 (0.94‐2.06)	1.37 (0.92‐2.05)	‐	‐
Persistent symptoms vs none	1.41 (1.04‐1.92)*	1.35 (0.98‐1.85)	0.94 (0.57‐1.56)	0.92 (0.55‐1.53)	2.36 (1.42‐3.92)*	1.99 (1.17‐3.40)*	2.03 (1.06‐3.87)*	1.97 (1.02‐3.83)*	‐	‐
Age of psychological distress assessment										
Ages 45‐54 years	0.74 (0.33‐1.62)	0.77 (0.35‐1.70)	0.99 (0.82‐1.18)	0.97 (0.81‐1.17)	1.35 (0.97‐1.86)	1.45 (0.99 (2.12)	1.14 (0.97‐1.34)	1.13 (0.96‐1.33)	‐	‐
Ages 55‐64 years	1.51 (1.27‐1.80)*	1.31 (1.07‐1.60)*	1.22 (1.04‐1.44)*	1.21 (1.03‐1.42)*	1.35 (1.14‐1.60)*	1.30 (1.09‐1.54)*	1.35 (1.14‐1.59)*	1.37 (1.16‐1.62)*	‐	‐
Ages 65‐75 years	1.22 (1.09‐1.36)*	1.20 (1.06‐1.35)*	1.42 (1.23‐1.65)*	1.41 (1.22‐1.64)*	1.40 (1.11‐1.77)*	1.40 (1.09‐1.80)*	1.26 (1.07‐1.48)*	1.26 (1.06‐1.49)*	‐	‐

*Note*: Associations with cognitive trajectories were examined using linear mixed models with random slope and intercept, with time coded in years and centered on baseline date to improve model stability and interpretability. Adj1: Age and sex. Adj2: Age, sex, marital (and cohabitation) status, education level, occupational social class, long‐term health conditions, smoking status, alcohol consumption, physical activity, baseline fluid cognitive level (ELSA ‐ fluid cognitive outcomes only) and childhood cognition (NCDS and NSHD ‐ fluid cognitive outcomes only).

Abbreviations: CAPS, Caerphilly Prospective Study; CI, confidence interval; ELSA, English Longitudinal Study of Ageing; NCDS, National Child Development Study; NSHD, National Survey of Health and Development; OR, odds ratio; WHII, Whitehall II.

Second, we examined associations between psychological distress and subsequent general fluid cognitive change using linear mixed models, based on interactions between psychological distress and time, in ELSA, NSHD, and WHII (Table 3; Figure S4; Tables S5 and S6). We observed decline in cognition over time in all three studies (ELSA: β = −0.04 [−0.04; ‐0.04]); NSHD (*β* = −0.01 [−0.01‐0.004]); WHII (β = −0.01 [−0.01‐0.01]; Table 3). When data were pooled across studies, baseline psychological distress, including clinically significant distress, was not associated with subsequent cognitive trajectories. In ELSA, persistent (*β* = −0.01 [−0.02; −0.002]), but not intermittent distress was associated with cognitive decline, but this was not observed in other[Fig alz71093-fig-0001] studies.

### 3.2 Psychological distress and dementia

**FIGURE 2:**
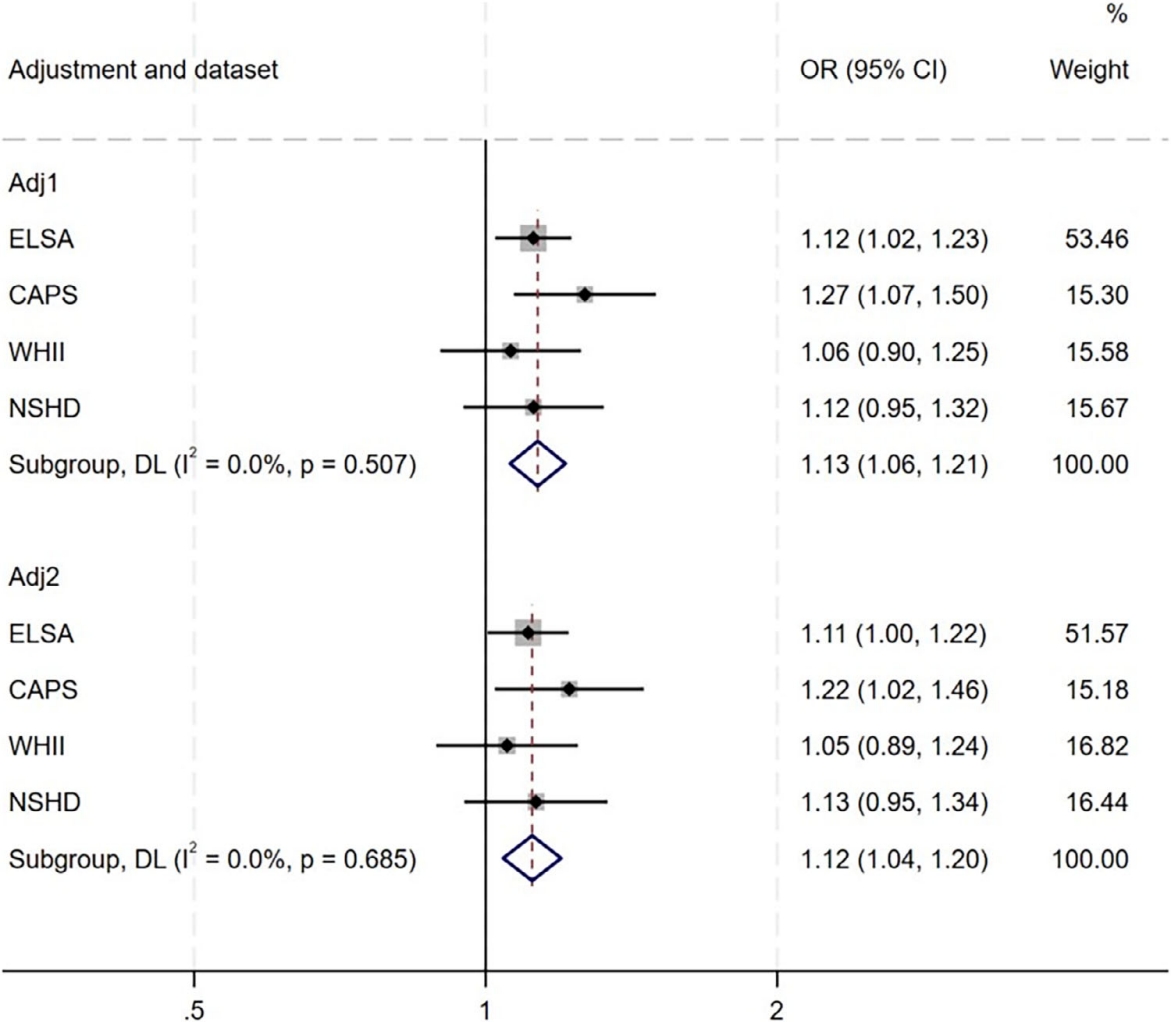
Pooled association between baseline continuous psychological distress and subsequent dementia. adj1, partially adjusted for age and sex; adj2, fully adjusted for covariates; CAPS, Caerphilly Prospective Study; CI, confidence interval; ELSA, English Longitudinal Study of Ageing; NSHD, National Survey of Health and Development; OR, odds ratio; WHII, Whitehall II.

**FIGURE 3:**
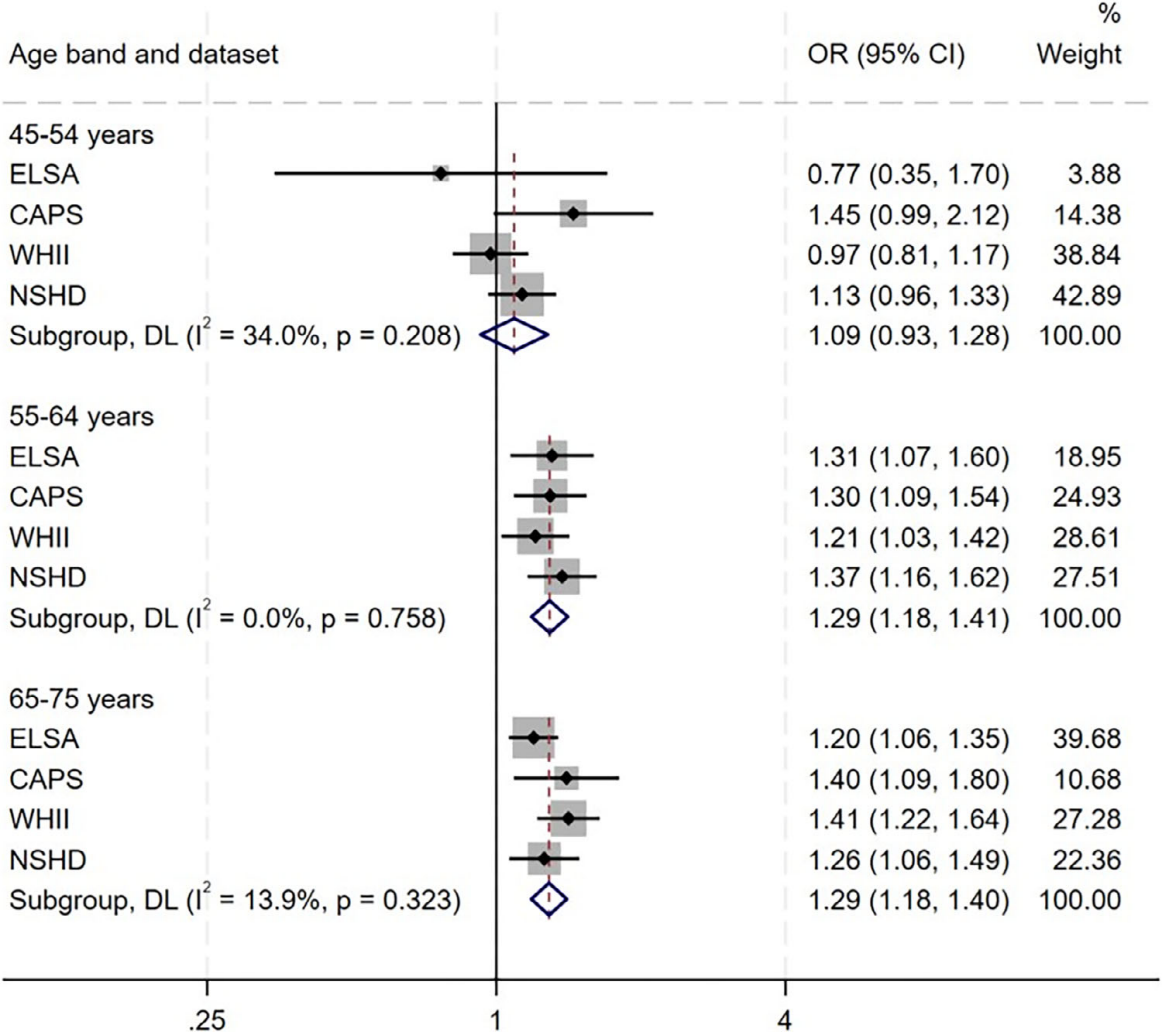
Pooled association between psychological distress and dementia by age of psychological distress assessment. CAPS, Caerphilly Prospective Study; CI, confidence interval; ELSA, English Longitudinal Study of Ageing; NSHD, National Survey of Health and Development; OR, odds ratio; WHII, Whitehall II.

Third, we examined associations between psychological distress and subsequent dementia in CAPS, ELSA, WHII, and NSHD (Table 3; Figures 2 and 3; Figure S5–S7). Pooling estimates across studies, we found evidence of an association between baseline psychological distress and subsequent dementia (fully adjusted odds ratio [OR] = 1.1 [1.0; 1.2]; *I* ^2^ = 0%). This was also observed for clinically significant symptoms (fully adjusted OR = 1.3 [1.1; 1.5; *I* ^2^ = 0%]). Both persistent (OR = 1.4 [1.0; 2.0]; *I* ^2^ = 44%) and intermittent distress were associated with subsequent dementia (OR = 1.3 [1.0; 1.7]; *I* ^2^ = 40%). We also examined variation in associations across the following age bands, where estimates could be pooled across studies: ages 45‐54; 55‐64; and 65‐75 years. Pooled associations between psychological distress and dementia were found when psychological distress was assessed at ages 65‐75 years (OR: 1.3 [1.2; 1.4]; *I* ^2^ = 14%) and 55‐64 years (OR: 1.3 [1.2; 1.4]; *I* ^2^ = 0%), but not at ages 45‐54 years (OR: 1.1 [0.9; 1.3]; *I* ^2^ = 34%).

### 3.3 Sensitivity analysis and subgroup differences

Patterns of association were similar when examining depression and anxiety symptoms individually (Figures S3 and S7), and in analyses restricted to studies using GHQ measures of psychological distress (Figures S8 and S9). Associations with cognitive change remained consistent in sensitivity analyses in which interactions between covariates and time were included in linear mixed models (Table S6). We did not observe effect modification by sex, education level, or occupational social class. We found no evidence of sex × psychological distress × time interactions in linear mixed models (Table S7), suggesting that associations between distress and cognitive change do not vary by sex. Similar patterns of association were found using Cox regression models to examine dementia outcomes in ELSA and WHII, where information on timing of dementia diagnosis was available. In ELSA, baseline psychological distress was associated with subsequent dementia (fully adjusted hazard ratio [HR]: 1.2 [1.1; 1.3]), and consistent with logistic regression models, baseline distress was not associated with dementia in WHII (HR: 1.0 [0.9; 1.2]). Associations remained in sensitivity analyses focused only on CAPS, NSHD, and WHII, where the GHQ was used to assess psychological distress (Figures S8 and 9).

## 4 DISCUSSION

### 4.1 Summary and meaning of findings

In this multi‐cohort study, psychological distress was associated with subsequent dementia and lower fluid cognitive level, but not with cognitive decline over time. These findings build on previous work by demonstrating that associations between psychological distress and both cognitive level and dementia risk are apparent across multiple UK cohorts using different measurement approaches. Observed associations despite differences in design and measurement strengthens evidence that psychological distress, even when transient or subclinical, is associated with poorer cognitive health in later life.

Previous research has mainly focused on depression, with fewer studies examining broader psychological distress. Our findings align with Finnish and Swedish population‐based studies that identified associations between psychological distress and dementia, despite their use of single‐item^10^ and non‐validated distress indicators.^6^ Similarly, findings from the Health and Retirement Study reported associations between distress and dementia over shorter follow‐up.^8^

We found associations across the full range of psychological distress symptoms, including clinically significant symptoms, and broader and subclinical aspects. This aligns with evidence that mild and subsyndromal depression are associated with dementia.^17^, ^18^ Fewer studies have examined severity or persistence in broader psychological distress. In a Japanese cohort, associations with dementia were found for both moderate and serious distress for those with lower baseline cognitive functioning, whereas for those with higher functioning, associations were only found for serious distress.^5^ We found that both intermittently and persistently high distress were associated with adverse cognitive outcomes, which could have implications for cognitive health at a population level, given the high prevalence of mild, intermittent distress symptoms across the life course.^34^ Few previous studies have examined symptom persistence in broader psychological distress, although a cohort study of women in Gothenberg reported a dose‐response relationship by distress persistence.^10^

In sensitivity analysis, depression and anxiety symptoms showed associations with poorer cognitive outcomes. This may reflect the substantial overlap and comorbidity between these symptoms,^35^ although, previous evidence on anxiety has been mixed, with several studies showing longitudinal links with dementia,^16^, ^36^, ^37^, ^38^, ^39^ while others did not observe associations.^40^, ^41^ Our findings therefore contribute to emerging evidence that anxiety may have relevance for dementia risk estimation, supporting consideration of this domain in future updates of dementia prevention frameworks.

We did not find evidence of variation by sex, education, or occupational class. This contrasts a Norwegian study reporting longstanding associations between depression and anxiety symptoms and dementia in women, whereas for men, associations were only found ∼11 years before, more consistent with reverse causality.^16^

The mechanisms underlying observed associations remain uncertain. Psychological distress could influence cognition through pathways such as reduced cognitive and brain reserve,^42^ and those involving chronic stress.^43^ Psychological distress is also linked with systemic inflammation,^44^ which is implicated in the pathophysiology of dementia.^45^ Psychological distress is also associated with poor cardiometabolic health^46^ and negative health behaviors,^47^ which have been identified as major risk factors for dementia.^4^ Residual confounding by shared genetic and environmental factors also remains possible.^15^

Reverse directionality is another possible explanation, given that dementia neuropathology develops over decades before clinical onset.^48^, ^49^ Psychiatric symptoms observed closer to dementia diagnosis could therefore reflect underlying neuropathology, rather than causal risk factors. In our study, associations with dementia were observed when psychological distress was assessed between ages 55‐64 and 65‐75 years, but not at age 45—54 years. This pattern aligns with findings from the FINRISK cohort^6^ and studies of late‐life depression showing stronger associations in later life and closer to dementia onset.^50^, ^51^, ^52^ Taken together, findings suggest that reverse directionality may partly contribute to associations between psychological distress and dementia.

However, longstanding associations with depression reported in several studies,^6^, ^7^, ^16^ and the inclusion of mid‐life depression in recent Lancet dementia commission updates,^4^ indicate that reverse causality is unlikely to fully account for observed associations. Further, in a Norwegian cohort, stronger associations with dementia were found for psychological distress assessed in early mid‐life compared to later mid‐life,^7^ indicating potential long‐term effects for some individuals.

Findings could have implications for clinical practice and early detection. In particular, if psychological distress symptoms reflect emerging neuropathology, this could facilitate earlier identification of individuals at increased risk of poor cognitive outcomes at an earlier stage, supporting clinical monitoring and appropriate referral pathways.

### 4.2 Strengths and limitations

Strengths of the study include the multi‐cohort approach involving five UK longitudinal studies, allowing a large overall sample size. Included studies had long follow‐up periods, with repeated measures of psychological distress and cognitive outcomes over many years, and rich information on a range of relevant sociodemographic and health‐related covariates.

Several limitations should be noted. First, although we adjusted for a range of sociodemographic and health‐related factors conceptualized as confounders, it is possible that some of these may lie on the causal pathway between psychological distress and cognitive outcomes. Interpretation of both partially and fully adjusted models therefore provides a more complete understanding of these relationships.

Second, despite efforts to harmonize exposures, outcomes, and covariates, it remains possible that heterogeneity between studies could reflect measurement differences. Measures of psychological distress varied in scope, although all have demonstrated validity as indicators of distress, and associations remained similar in sensitivity analyses restricted to studies using GHQ measures of psychological distress. Cognitive tests and dementia ascertainment also varied between studies (e.g., inclusion of people with cognitive impairment without dementia in CAPS; and the use of AD8 to capture very mild dementia symptoms in NSHD). These differences should be considered when interpreting results, although modest heterogeneity across dementia estimates supported pooled analyses. In addition, given that z‐scores for cognitive outcomes were derived within each study, we note that comparable effect sizes do not necessarily imply equivalent absolute differences in cognitive level or cognitive change across studies.

Third, patterns of missing psychological distress data may have influenced findings. Individuals with higher distress levels may be less likely to participate, and differing levels and patterns of missingness across studies could contribute to between‐study variation. Variation between studies could also reflect differences in study settings, follow‐up duration, and ages at baseline assessments. In addition, CAPS and NSHD lacked information on timing of dementia diagnosis, and CAPS included men only. Not all studies had repeat cognitive assessments or dementia measures, limiting analyses focused on cognitive change and dementia to a subset of studies.

Fourth, logistic regression was used in analyses focused on dementia to maximize comparability across studies, although this approach does not account for variation in follow‐up or mortality. Nonetheless, findings were consistent in sensitivity analyses using Cox regression in ELSA and WHII, where information on timing of dementia outcomes was available.

Finally, examining associations between psychological distress and cognitive outcomes across different datasets with differing but conceptually related measures of psychological distress and cognition could also be considered a strength, as it supports the robustness of findings through replication. It is increasingly recognized that findings conceptually replicating across comparable but varying designs and measures can strengthen inference more than exact replication approaches.

## 5 CONCLUSION

Applying a multi‐cohort approach, we found that psychological distress was associated with poorer subsequent fluid cognitive level and dementia, although not with cognitive trajectories over time. Associations were not limited to persistent, clinically significant distress, but were found across the full range of psychological distress symptoms, including intermittent distress. Stronger associations at older ages, combined with previous evidence on temporality, indicate that reverse causality may contribute, but is unlikely to fully explain these findings.

Further research is needed to distinguish causal effects from shared underlying risk factors, and to examine dementia risk in relation to a wider range of psychiatric symptoms. Distinguishing longstanding from prodromal associations will be important for informing prevention strategies. For symptoms observed closer to dementia onset, findings could help in earlier identification of high‐risk groups for poor cognitive outcomes, who may benefit from enhanced monitoring for cognitive and functional symptoms, and targeted clinical support.

## CONFLICT OF INTEREST STATEMENT

The authors have declared that there are no conflicts of interest in relation to the subject of this study. Author disclosures are available in the Supporting Information.

## CONSENT STATEMENT

All human participants within each dataset had provided informed consent to take part.

## Supporting information



Supporting Information

Supporting Information
